# Precise Partitioning of Metallic Single-Wall Carbon Nanotubes and Enantiomers through Aqueous Two-Phase Extraction

**DOI:** 10.1021/acsnano.5c00025

**Published:** 2025-04-03

**Authors:** Han Li, Ming Zheng, Jeffrey A. Fagan

**Affiliations:** † Department of Mechanical and Materials Engineering, University of Turku, Turku FI-20014, Finland; ‡ Turku Collegium for Science, Medicine and Technology, University of Turku, Turku FI-20014, Finland; § Materials Science and Engineering Division, National Institute of Standards and Technology, Gaithersburg, Maryland 20899, United States

**Keywords:** metallic SWCNT, enantiomer sorting, surfactant wrapping, partition coefficient change condition, cooperativity

## Abstract

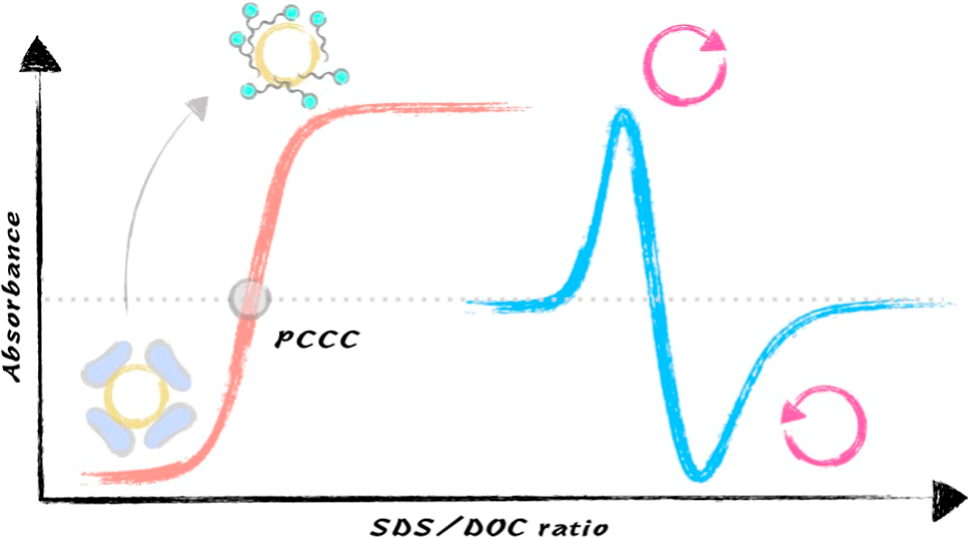

Separation of single-chirality single-wall carbon nanotubes (SWCNTs) and their enantiomers holds significant potential for materials science and various applications but challenges in scalability and precision persist. In this study, we introduce a systematic approach to identify separation conditions for metallic SWCNTs in aqueous two-phase extraction (ATPE), precisely identifying improved conditions for isolating multiple armchair and chiral (*n*,*m*) species. We quantify these conditions by determining partition coefficient change condition (PCCC) values for both binary and ternary surfactant combinations. This information enables optimization for efficient separation of high-purity armchair nanotubes such as (6,6), (7,7), (8,8) and (9,9), and for isolation of enantiomeric nonarmchair nanotubes, including challenging metallic species such as the (8,5), (7,4), (9,3), (10,4) and (10,7). Lastly, separated single (*n*,*m*) populations are reseparated in ATPE at precise steps in both binary and ternary surfactant mixtures to resolve their enantiomers, extracting information on the underlying mechanism of metallic SWCNT ATPE and highlighting the utility of sodium cholate for achieving single enantiomer level separations.

Single-wall carbon nanotubes (SWCNTs), known for their unique optical and electronic properties linked intricately to their atomic structures, have consistently captivated interest and attention as one of the most intensively studied nanomaterials over the past three decades.^1^ Each species of SWCNT has a unique lattice structure describable by a set of integers, (*n*,*m*), derived from the chiral vector, *C*_h_ = *n*a_1_ + *m*a_2_, specifying the points brought together to create the cylindrical SWCNT form on a notional hexagonal graphene lattice plane (a_1_ and a_2_ are the unitary lattice vectors along the zigzag and armchair lattice directions).^2^ SWCNTs are broadly classifiable into two electronic types based on their (*n*,*m*) integers (chirality); mod3(*n*-*m* = 1 or 2) are semiconducting with an ≈0.5 to 1 eV bandgap, while mod3(*n*–*m* = 0) are metallic (only *n* = *m* SWCNTs are expected to be truly metallic, all others are predicted to be very small bandgap semiconductors, typically with a bandgap less than thermal energy (*k*_B_*T*)).^3,4^ They are also specifiable with respect to symmetry in the graphene sheet, with nonchiral armchair (*n* = *m*) and zigzag (*m* = 0) species having twists of 30 and 0°, respectively, and chiral (*n* ≠ *m* and *m* ≠ 0) values in between.^2^ Only chiral SWCNTs have enantiomers, with synthetic methods thought to produce racemic mixtures of left-handed and right-handed spiral twists.^5^ This fact is important, as handedness generates optical activity and affects interactions including binding by chiral adsorbates and handed phenomena such as spin transfer.^6^

Despite significant advancements in the synthesis and application of SWCNTs, challenges remain in achieving high-precision and scalable separations of SWCNT populations based on their electronic nature, (n,m) species (chirality) and enantiomeric twist. Methods such as density gradient ultracentrifugation (DGU),^7,8^ gel chromatography (GC),^9,10^ and polymer wrapping (CP)^11,12^ have been employed to separate SWCNTs by their diameter, electronic properties, or chirality, however, these techniques have limited applicability for metallic SWCNTs.^13^ Despite significant effort, literature results note few separated metallic (*n*,*m*) species,^14−19^ typically extracted heroically due to low yields or not easily scaled processes. Recent developments for metallic and many semiconducting SWCNT (*n*,*m*)s have instead shifted focus toward an aqueous two-phase extraction (ATPE) separation method.^20−25^ This technique is grounded on affinity-based selection of solutes between two spontaneously self-separating aqueous phases, which in most implementations consist of polyethylene glycol (PEG) and dextran (DX) polymers.^26^ Mixtures of these polymers become inherently self-separating for concentrations above their binodal point and spontaneously phase separate into hydrophobic (PEG rich) top and hydrophilic (DX rich) bottom phases. Any dispersed solutes, such as SWCNTs, sensitively and selectively distribute themselves between the two phases primarily on the basis of their solvation energy in each of the two phases.^27^ For SWCNTs, each (*n*,*m*)’s (and enantiomer’s) solvation energy in the separating polymer phases are in turn associated with their interfacial coating. Because the interfacial adsorbed layer structure and chemistry is controllable by dispersant selection, structure, or concentration (in the case of competing cosurfactants), ATPE offers a promising route for specified separation that is simple, scalable, and effective in sorting SWCNTs based on subtle differences in their physical and chemical properties.^26,27^

While ATPE offers advantages to comparative separation methods, it is not without its limitations. The precision of ATPE in sorting SWCNTs by chirality and electronic types can be inconsistent due to the complexity of environmental effects,^26^ its inherent nature as a multistep process,^28^ aggregation effects under some conditions,^26^ and remaining incompleteness in our understanding of dispersant or cosurfactant interactions for the surface of different (*n*,*m*) SWCNTs.^29,30^ Although previous enhancements, such as pH modulation,^20,21^ have refined the ATPE process by offering better control, the fundamental challenge of specifying and controlling the sorting outcomes with high accuracy persists. For surfactant-controlled ATPE such as in this contribution, a key factor is measuring and determining the concentrations of surfactants at which a specific SWCNT (*n*,*m*) switches from partition in one phase to selecting partition in the other.^29^ We call the surfactant concentration values at which this selection change occurs the partition coefficient change condition (PCCC).^31^ Orthogonal measurements have shown, in studied cases on semiconducting species, that each species’ (and enantiomer’s) PCCC value physically corresponds to a sudden change in the composition of the surfactant adsorbed surfactant layer on the nanotube; this change then drives the switch in polymer phase selection in ATPE.^32,33^ Historically, these PCCC values were identified from optical spectroscopy on separations of polydisperse SWCNT populations,^30^ which, while significantly advancing, lack precision for many (*n*,*m*)s and are considerably tedious for broad investigative screening. Moreover, metallic SWCNTs, are generally present at lesser concentrations and have weaker optical features than semiconducting species, and as such have been particularly challenging to study by this methodology. More recently, Sims et al.,^34^ has significantly advanced our ability to determine PCCC values, including the partition behavior of (6,5) enantiomers,^23^ using a fluorescence-based method that does not require an ATPE separation. However, this approach inherently cannot be applied to metallic SWCNTs as they do not fluoresce.

Addressing these challenges, this paper investigates the use of surfactant-controlled ATPE to sort metallic SWCNTs and their enantiomers by performing separations twice, once to generate highly pure populations, and the second time to characterize their partition behavior. By this methodology we can obtain precise PCCC values for each of the single-chirality metallic SWCNT species and their enantiomers at actual sorting conditions, with quantification possible by assuming a simple model based on the Hill equation. In addition to determining and comparing precise values in the most commonly utilized ATPE cosurfactant system, the same separated parent stocks are also utilizable to explore other surfactant systems that may optimize the sorting process. We demonstrate this by the first determination of PCCC values for multiple metallic species in a three-component surfactant competition. Moreover, we show that this system enables particularly efficient separation of high-purity armchair nanotubes including the (6,6), (7,7), (8,8) and (9,9). Our findings also demonstrate that inclusion of sodium cholate (SC) notably improves the sorting efficiency of nonarmchair metallic SWCNT enantiomers (e.g., (8,5), (7,4), and (10,7)), addressing some of the most challenging aspects of SWCNT separation. Together these observed changes in the PCCC in binary and ternary surfactant systems provide new insights into the dynamics of surfactant–nanotube interactions.

## Results

### PCCC in a Real ATPE Process

To enable precise identification of PCCC values we employ a methodology that starts with metallic (*n*,*m*) SWCNT samples enriched by a prior round of ATPE separations. The basic requirement for a presorted sample is that the first order optical transition (M_11_) peaks for the target species are distinct and not significantly overlapped to ensure clear identification of partition. For a broad study, SWCNT soot samples produced by different synthesis methods were utilized to obtain a range of presorted metallic species. This is necessary because each commercially available soot contains a different range of average diameters and relative abundances of the possible (*n*,*m*) species, with no single soot adequately containing all of the species investigated in this effort. Further details of sources and preseparation are provided in the Supporting Information as Figure S1.

**Figure 1:**
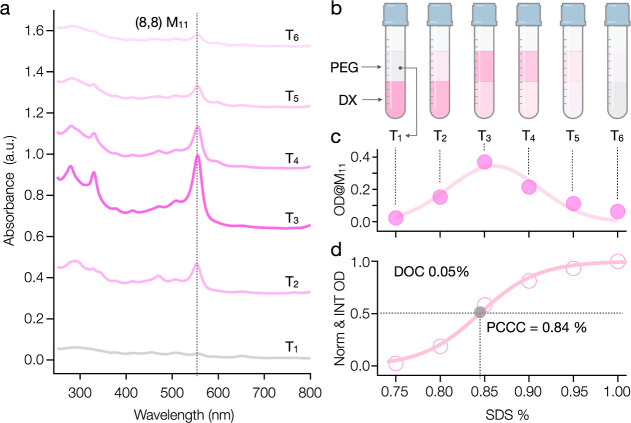
Quantitative analysis of SWCNT partitioning in ATPE. (a) Absorption spectra of presorted (8,8) SWCNTs across successive ATPE extractions. The changes in M_11_ peak intensity demonstrate the change of (8,8) concentration in the top phase. (b) Schematic representation of the ATPE setup across six extraction steps (T1 to T6). (c) Optical density (OD) values at the M_11_ peak for (8,8) SWCNTs measured in the top phase of each ATPE step, plotted against the corresponding SDS concentrations. (d) Integrated and normalized OD curve, depicting the sigmoidal transition of (8,8) SWCNTs from the bottom to the top phase. The PCCC, determined as 8.4 g/L (0.84%) SDS for 0.5 g/L (0.05%) DOC, is fit by the Hill equation, indicating the critical transition point. All absorbance measurements were conducted with a mimic top phase used to subtract the minimal absorbance contributions from the polymers, water and cuvette. The spectra are offset for clearer comparison.

An example of the characterization methodology for the ATPE sorting process used in this contribution is shown in Figure 1 for the (8,8) SWCNT species. Note that the optical transitions used to assign the (*n*,*m*) species of metallic SWCNTs in this work are obtained from previous studies,^35−38^ which are also comprehensively summarized by Hároz et al.^39^ In this methodology, the composition of the ATPE system is set by direct construction at the first surfactant concentration set of interest, centrifugation is applied to drive complete resolution of the two polymer phases, and the top phase is completely removed for SWCNT concentration assessment via ultraviolet–visible-near-infrared (UV–vis-NIR) absorbance spectroscopy. A true mimic top phase, containing both polymers and surfactants, but without SWCNTs, is then added to the retained bottom phase to increment the surfactant concentrations of the overall volume assuming volumetric weighting. For the example in Figure 1, the concentration of sodium deoxycholate (DOC) was maintained at 0.5 g/L (0.05% (mass/volume)), while the concentration of sodium dodecyl sulfate (SDS) was incrementally increased across five partition steps from (7.5 g/L, 0.75%) to 10 g/L (1%) by a uniform interval of 0.5 g/L (0.05%) for each step. Note that the minimal increment of 0.05% SDS was deliberately chosen throughout all the sorting processes in this work to standardize the procedure and to minimize experimental errors and uncertainties. This approach broadly ensures that a sufficient quantity of SWCNTs shift to the top phase for accurate concentration measurement by absorbance spectroscopy at each step, reducing the variability that might arise from smaller SDS adjustments.

As the SDS concentration (and ratio to DOC) is increased at each step, the nanoscale adsorbed surfactant layer on each nanotube re-equilibrates, changing or not in terms of the dominant surfactant comprising the layer depending on the (*n*,*m*) species. Consequently, nanotubes coated predominantly with SDS tend to shift to the PEG-rich top phase, whereas those primarily coated with DOC remain in the DX-rich bottom phase. This results in some (*n*,*m*)s shifting their partition from the bottom to the top phase, as shown in Figure 1b. The enriched (8,8) fraction displayed the clear M_11_ peak (at 555 nm) throughout the entire extraction process (see Figure 1a), allowing us to obtain the optical density (OD) at M_11_ and concentration of nanotubes in each top phase fraction (see Figure 1c, T_1_ to T_6_). We use only the M_11_ feature for quantification because the absorbance spectra of metallic SWCNTs at energies above their lowest order direct interband transition, i.e., M_11_, can be complex, with multiple peak features.^39^ Other than for confirming assignment of the metallic species being observed, we thus do not use these features to track the concentration changes with ATPE partition. Fortunately, with preseparation, the M_11_ features for the metallic SWCNTs used in this study are strong relative to non (*n*,*m*) specific extinction, and integration over this feature adds little uncertainty to PCCC determination. Use of initially enriched samples for these studies is thus important for assigning OD to the nanotube of interest without requiring deconvolution of contributions or complex analysis.

In Figure 1c, the OD is plotted for each fraction at the specific step of extraction, whereas Figure 1d presents the cumulative portion of (8,8) nanotubes that have moved to the top phase across all steps. By integrating and normalizing the OD data combined from all fractions, we generate a sigmoidal curve, capturing the transition of (8,8) nanotubes from the bottom to the top phase during the ATPE process (see Figure 1d). Note that surfactant concentrations are reported in apparent mass/volume percent in the text and figures below for consistency with SWCNT separation literature.

Following the work by Oh et al.^40^ on surfactant binding affinities, we apply the Hill equation to quantitatively analyze the observed sigmoidal partition behavior (Figure 1d), focusing particularly on the PCCC, which we define as the midpoint of the sigmoidal curve of the integrated optical density (OD_norm & INT_)
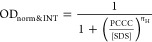
1

This critical point, precisely identified at an SDS concentration of 8.4 g/L (0.84%), which reflects an SDS/DOC mass ratio of 16.8:1 and a molar ratio of 24.2:1, indicates the condition at which the (8,8) nanotubes transition from preferring bottom to top phase partitioning. The Hill coefficient (*n*_H_ = 25.6) reflects the curve’s steepness near this midpoint, and is interpretable as a signature of strong positive cooperativity in the partition-controlling surfactant adsorption to the nanotube surface.^41^ Note that these values are based on single measurements as examples. Comprehensive averages and uncertainties from multiple measurements will be provided for more precise separations described subsequently. As discussed later, it is unlikely that the SDS surfactant is the source of the observed cooperativity, and we instead attribute the observation of apparent cooperativity to the simultaneous replacement of a large number of cooperatively adsorbed DOC molecules by a large number of SDS molecules at the critical transition point.

**Figure 2:**
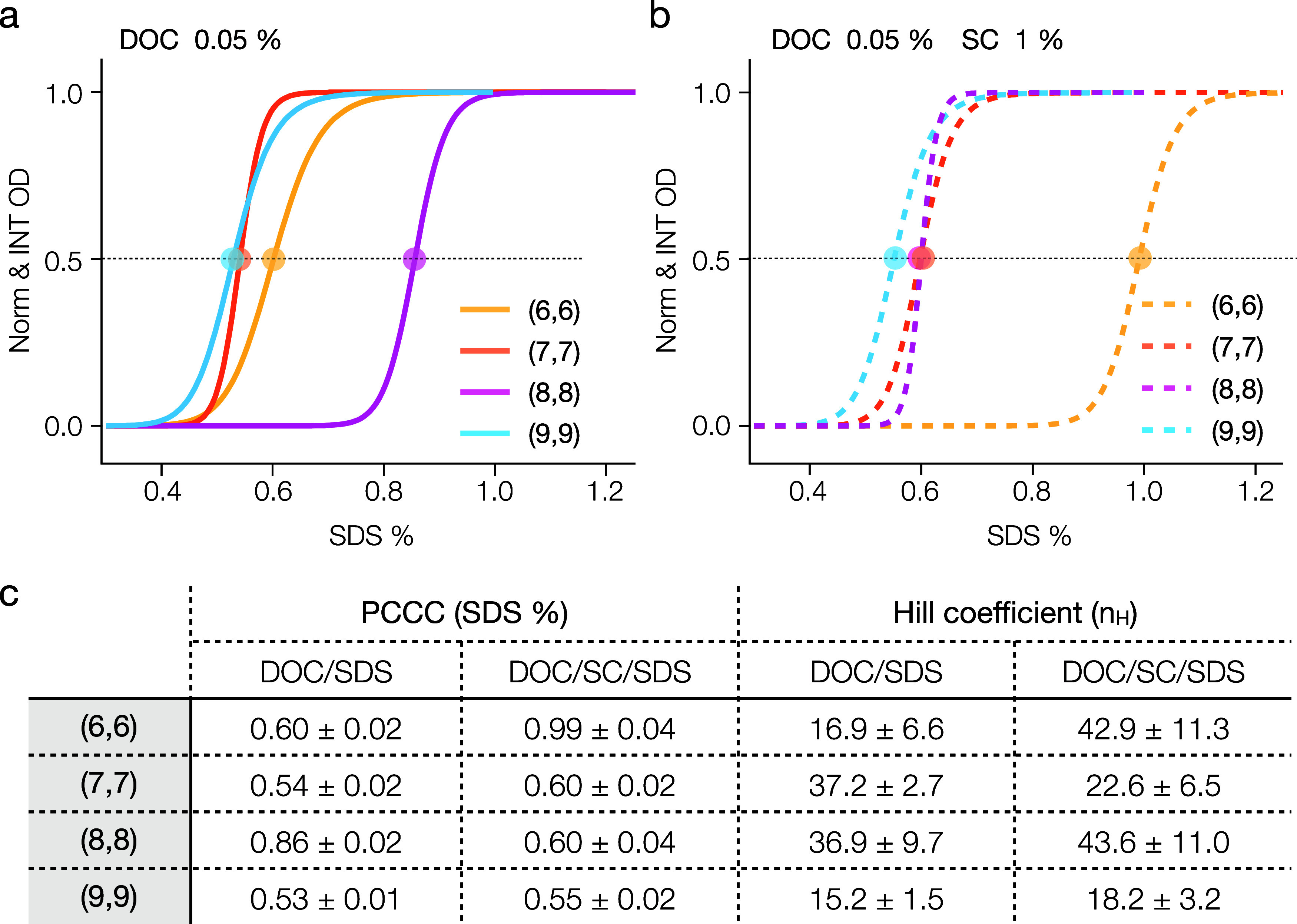
Impact of binary and ternary cosurfactant systems on the PCCC of armchair SWCNTs. (a) The partition curves for four armchair SWCNTs ((6,6), (7,7), (8,8), and (9,9)) using the DOC/SDS binary-cosurfactant system show diameter-dependent PCCC ordering, with (8,8) as the exception. (b) The partition curves in the DOC/SC/SDS ternary-cosurfactant system illustrate a normalized PCCC for (8,8) and an altered partition for (6,6). The corresponding PCCC values and Hill coefficients for each system are presented in (c).

Following the same procedure, we determined PCCC values for other armchair SWCNTs including the (6,6), (7,7) and (9,9) species. Figure 2a illustrates the partition curves for all four armchair-type nanotubes. The absorption spectra for each fraction are reported in the Supporting Information (Figure S2). We can see that for (6,6) (7,7) and (9,9) in DOC/SDS binary-cosurfactant mixtures there is a general trend of an increasing PCCC for decreasing SWCNT diameter in the sorting order, which is consistent with prior reports.^22,25^ Counter to this trend, the (8,8) is a clear outlier presenting a greater than would be expected PCCC value, however, this is also in accordance with previous reports.^15,29^ The improved precision PCCC values of this contribution are listed in the Table within Figure 2c.

### Extension to Three-Surfactant ATPE Systems for Sorting Armchair SWCNTs.

While DOC/SDS surfactant ATPE PCCC values for some metallic species have been previously reported,^29^ albeit at lower precision than this work, PCCC values in other multiple-surfactant competition ATPE systems are less available. As such, we used the same methodology to determine PCCC values for a three-surfactant combination of DOC, SDS and SC that has been reported as being particularly resolving for semiconducting SWCNT species.^21,23^ This system adds a constant 10 g/L (1%) SC to the same (constant) DOC concentration (0.05%) while varying the SDS concentration (DOC/SC/SDS ternary-cosurfactant system). PCCC results for equivalently conducted ATPE separations as the DOC/SDS competition are reported through corresponding sigmoidal curves in Figure 2b and PCCC values in the Figure 2c table. Detailed absorption spectra for each fractionation step are reported in the Supporting Information (Figure S3). Although SC is a much more weakly adsorbing surfactant than DOC, inclusion of SC in the ATPE system results in significant changes to both the PCCC values and overall sorting order.^22^ Significantly, we observe that the PCCC of (8,8) jumps to a notably lesser SDS concentration, 0.6%, that is almost overlapped with the value for the (7,7), but becoming consistent with the near monotonic (*n*,*m*) extraction order with decreasing SWCNT diameter observed for most species.^25^ Interestingly, despite most species also extracting in decreasing diameter order in the ternary cosurfactant system, the (6,6) becomes an outlier, exhibiting a surprisingly large shift in its PCCC value. Its PCCC increases dramatically to ≈1% SDS and becomes distinctly separated from those of the other three armchair tubes. The Hill coefficients for all four SWCNT (*n,m*)s in both binary and ternary cosurfactant systems are listed in Figure 2c. The addition of SC clearly affects the competitive adsorption of surfactants differently across the SWCNT species. Next, we further investigate how the two cosurfactant systems might influence other aspects of ATPE performance on the metallic SWCNT sorting.

### Characterization of Binary and Ternary Cosurfactant System Effects on ATPE of Nonarmchair Versus Armchair SWCNTs

An advantage of the ATPE technique is that the surfactants used for controlling partitioning can be easily specified for any given separation step, i.e., pragmatically one can switch back and forth between DOC/SDS and DOC/SC/SDS determined separations to optimize their separation to target a specific (*n*,*m*), but only if differentiating conditions are known. Thus, identification of conditions that distinctly differentiate (*n*,*m*)s with similar PCCC values in DOC/SDS ATPE by DOC/SC/SDS ATPE is of particular interest. Partition curves such as those of this work enable quantitative comparison and analysis of PCCCs in the different cosurfactant systems and help selection of easier conditions for isolating difficult species, especially nonarmchair metallic tubes, which we now focus on.

Nonarmchair SWCNTs species, excepting the (7,4), are rarely described postseparation for several reasons. Chief among these is the lesser (and decreasing) abundance for nearer to zigzag species in SWCNTs synthesized by commercial synthetic processes,^42,43^ but other factors include PCCC values similar to those of other low abundance species, and, heuristically, significant lot to lot variation in the abundance of these species, e.g., we have observed the fraction of (8,5) to vary from readily detectable to nearly nonexistent in different batches of the same commercial product. A final factor is that, observationally, nonarmchair metallic species dedope from oxidative environments more slowly than armchair metallic SWCNTs; from an ATPE process standpoint this can result in a further down selection or discarding of their containing fraction due to contamination by apparently defective SWCNTs that partition similarly and have poor optical properties.

**Figure 3:**
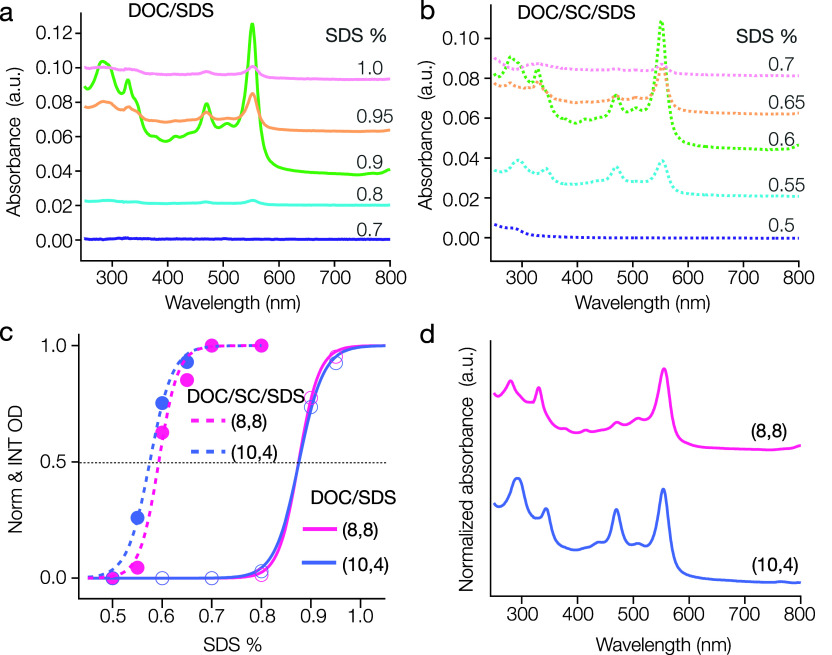
(a) Absorption spectra of enriched (8,8) with (10,4) sorting in the DOC/SDS system, with a fixed 0.05% DOC. SDS concentrations for each step are marked on the graph. (b) The absorption spectra of similar sorting steps in the DOC/SC/SDS system, with a fixed 0.05% DOC and 1% SC. Purified (10,4) can be separated at 0.55% SDS. (c) The distinct partition curves for (8,8) and (10,4) in the binary (solid line) and ternary (dashed line) systems respectively, showing a small gap that suggests potential for (10,4) separation. (d) Absorption spectra of the resolved (8,8) and (10,4) in 1% DOC postseparation. The spectra are offset for clearer comparison.

Figure 3a shows results for aliquots of an identical (8,8)-enriched sample, here focusing on both the (8,8) and the (same family) nonarmchair (10,4) species, sorted under either binary (DOC/SDS) or ternary (DOC/SC/SDS) cosurfactant conditions. The binary system used a fixed DOC concentration of 0.05%, whereas the ternary system used fixed SC (1%) and DOC (0.05%). Both systems employed a minimal SDS increment of 0.05% (Figure 3a,b). In addition to a shift in PCCC values for both species for the binary versus ternary systems, sufficient PCCC resolution to distinguish between the (8,8) and the nonarmchair SWCNT (10,4) impurity in the (8,8) enriched parent is observed.

The fitted partition curves in Figure 3c for (8,8) and (10,4) display highly overlapping partition conditions (PCCC_(8,8)_ = 0.86%, PCCC_(10,4)_ = 0.87%) in the DOC/SDS binary system, while in the DOC/SC/SDS ternary case a small, but more resolvable, gap is observed (PCCC_(8,8)_ = 0.60%, PCCC_(10,4)_ = 0.56%). This suggested the potential for obtaining a pure fraction of (10,4) by sequential separation utilizing both ATPE systems. Figure 3d presents the absorption spectra of (8,8) and (10,4) separated via this difference after transfer to a 1% DOC solution environment, demonstrating the resolving power of even small PCCC differences using ATPE as long as those differences are known. Note that the PCCC of (8,8) in the binary surfactant conditions, as extracted here, comes from a starting suspension with a relatively higher ratio of (10,4) compared to what is shown in Figure 1. Despite this difference, the PCCC values are closely matched, with uncertainties within ±0.02% SDS. The average values are presented in Figure 2c and Table S1 in the Supporting Information.

**Figure 4:**
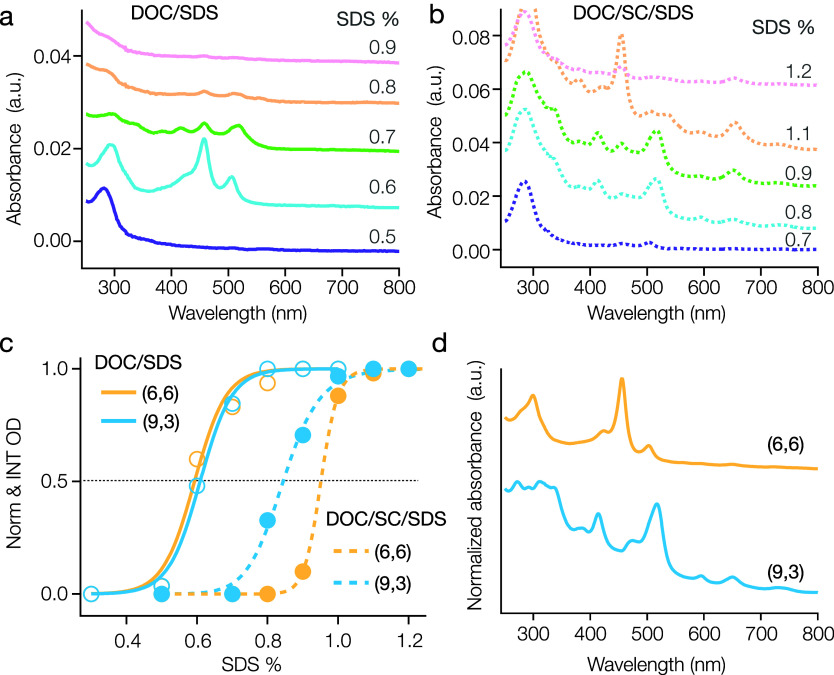
(a) Absorption spectra of enriched (6,6) with (9,3) in the DOC/SDS system with a fixed 0.05% DOC concentration with SDS concentration steps marked on the graph for each spectrum. (b) Similar absorption spectra for the DOC/SC/SDS system with fixed 0.05% DOC and 1% SC. (c) Partition curves for (6,6) and (9,3) in the binary system (solid lines) and the ternary system (dashed lines), demonstrating easier separation of (9,3) in the ternary case. (d) Normalized absorption spectra of the purified (6,6) and (9,3) in 1% DOC. The spectra are offset for clearer comparison.

A similar differentiation is observed for the (6,6) and (9,3) species, which likewise partition closely together in the binary surfactant competition, but with greater differentiation in the ternary-cosurfactant system. As shown in Figure 4, it is nearly impossible to separate all impurity (9,3) from (6,6) (PCCC_(6,6)_ = 0.60%, PCCC_(9,3)_ = 0.60%) in the DOC/SDS system, while with the addition of SC a strong differentiation is observed (PCCC_(6,6)_ = 0.99%, PCCC_(9,3)_ = 0.84%).

The characterization above clearly demonstrates that altering the surfactant competition can produce differentiating partition conditions for nonarmchair SWCNTs such as (10,4) and (9,3), even though these species are typically challenging to separate due to their scarce abundance, compared to armchair species, generally in commercially available raw SWCNT soot. The partition curves indicate that they can be more effectively purified and yielded using the ternary-cosurfactant system.

### Comparing Binary Versus Ternary Cosurfactant Systems for Metallic SWCNT Enantiomer Sorting

We further investigate the partition of enantiomers for nonarmchair SWCNTs in the different cosurfactant systems. It is known that (8,5) is normally a challenge to separate because its M_11_^+^ and M_11_^−^peaks (at 457 and 501 nm as measured in 1% DOC) are highly overlapped with the M_11_ peaks of (6,6) and (7,7) at 456 and 504 nm (in 1% DOC) respectively (see Figure S4). This directly complicates the purity assessment of (8,5) by either visual inspection or absorbance spectra during performance of the ATPE process, so in the lack of precise PCCC values it was difficult to judge how much SDS to use for discrimination of the species. Frustratingly, for the DOC/SDS ATPE the PCCC values between (8,5), (6,6) and (7,7) are fairly similar (Table S1), providing little resolution for distinguishing the (8,5). In our opinion only one plausible (8,5) fraction can be readily separated (see Figure S4). However, in the first section of this contribution we observed that we can effectively delay the PCCC of (6,6) to nearly 1% SDS by using the ternary- DOC/SC/SDS cosurfactant system (Figure 2b). This suggests a simplified ATPE sorting process that focuses only on distinguishing between the (8,5) and (7,7) in a last separation stage. Explicitly, by shifting the PCCC of (6,6) toward a higher SDS concentration, we are able to remove (6,6) as a major contaminant before focusing on separating (8,5) from (7,7). We then perform a follow-up extraction step to separate (8,5) from any remaining (7,7). When the M_11_^+^and M_11_^−^ peaks have comparable OD, it indicates that we have isolated predominantly (8,5). Although minor amounts of (7,7) cannot be fully ruled out, this combined approach enables significantly enhanced (8,5) purity in fewer separations compared to a DOC/SDS procedure alone.

**Figure 5:**
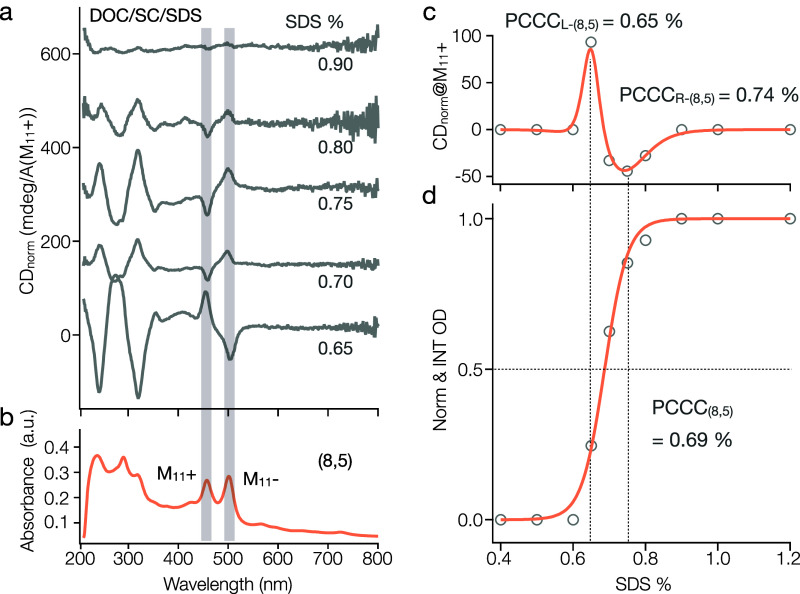
Enantiomer separation of (8,5) in the DOC/SC/SDS system. (a) CD spectra for (8,5) enantiomers at increasing SDS concentrations from 0.65% to 0.90%, normalized by the M_11_^+^ peak OD values from the absorption spectra. (b) A typical absorption spectrum of (8,5) showing both the M_11_^+^ and M_11_^−^ peak positions, corresponding to the plus and minus CD peaks in (a). (c) Normalized CD values at the M_11_^+^ are plotted against SDS concentration and fitted using a modified Hill equation in differentiated form, indicating the enantiomer PCCCs. (d) The corresponding partition curve for (8,5) demonstrates the single-chirality PCCC. All absorption and CD measures are conducted in 1% DOC in H_2_O. The spectra are offset for clearer comparison.

Moreover, the enhanced differentiation by the DOC/SC/SDS ATPE system not only results in more effective (8,5) sorting (see Figure S4c), but also increases the resolution of its enantiomers. As shown by normalized circular dichroism (CD) data in Figure 5a, four fractions of first left-, then right-, handed (8,5) SWCNTs can be separated by adjusting between 0.65% SDS to 0.8% SDS concentration in the ternary system. An incorrect analysis of the extraction in Figure 5a as a single component would imply an inconsistently broad PCCC transition compared to observations both above and in the literature. Such an inconsistency is resolved if the integrated absorbance-derived curve is instead attributed to two components, i.e., the two distinct twist enantiomers of the (8,5). The contributions of the two enantiomers can be resolved through their differences in optical activity. Note that twist assignment is by the theoretical framework proposed by Sato et al.,^5^ in which an enantiomer exhibiting a positive M_11_^+^ CD peak is assigned to be a left-handed SWCNT (L-SWCNT), while a negative M_11_^+^ peak indicates a right-handed SWCNT (R-SWCNT). Figure 5a illustrates that in the DOC/SC/SDS system, most of the L-(8,5) enantiomers initially partition to the top phase, followed by the R-(8,5) with comparatively lower purity. Using a differentiated form of the Hill equation, we fit the extracted CD values from the M_11_^+^ peaks of each fraction (as shown in Figure 5c; see Supporting Information for details). The peaks at SDS concentrations of 0.65% and 0.74% mark the partition points for L-(8,5) and R-(8,5) respectively, and the corresponding peak values at M_11_^+^ (+85.9 mdeg/A and −43.5 mdeg/A) further underscore the purity levels of both enantiomers. This approach allows us to precisely align the partition curve (shown in Figure 5d) and accurately determine the PCCC for each enantiomer.

Similar procedures have also been conducted on the sorting of larger diameter nonarmchair SWCNTs from other types of raw soot, for example (10,7), yielding analogous results. These results are detailed in Figures S5 and S6 in the Supporting Information.

**Figure 6:**
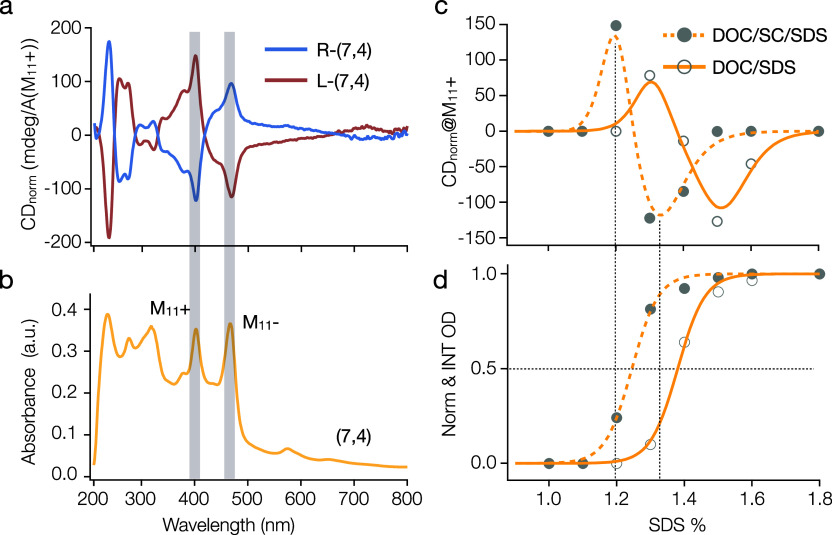
Enantiomer separation of (7,4) in binary and ternary cosurfactant systems. (a) Typical CD spectra showing the separation of L- and R-(7,4). (b) Absorption spectrum of (7,4), highlighting the M_11_^+^ and M_11_^−^ peak positions corresponding to the CD peaks in panel (a). (c) Normalized CD values from M_11_^+^ plotted against SDS concentration, illustrating the enantiomer separation trend and fitted using a modified Hill equation, indicating distinct PCCC for L- and R-(7,4). (d) Partition curves demonstrating the PCCC for (7,4), where the addition of SC lowers the necessary SDS concentration for separation. All absorption and CD measures are conducted in 1% DOC in H_2_O.

For more easily enriched nonarmchair species like (7,4), which is present in notable abundance in many of our commercially obtained small diameter SWCNT sources, we conducted a comparable analysis of enantiomer separation in both cosurfactant systems. Shown in Figure 6, a similar sorting procedure applied to (7,4) results in the isolation of both enantiomers. Figures 6a,b show the typical CD and absorption spectra of L- and R-(7,4) (spectra throughout the sorting process are in the Supporting Information Figure S7). The fitted PCCC curves for enantiomeric and single-chirality (7,4) are presented in Figure 6c,d, respectively. We found that the inclusion of SC in the system results in the partitioning of (7,4) at a reduced SDS concentration (Figure 6d, PCCC = 1.24% in the DOC/SC/SDS system and PCCC = 1.38% in the DOC/SDS system), aligning with our previous findings on (6,5).^23^Figure 6c illustrates that the enantiomer partition follows a similar trend, with L-(7,4) partitioning at a lower SDS concentration than R-(7,4). Moreover, the ternary cosurfactant system clearly enhances the enantiomeric purity for both L- and R-(7,4). Although reports of CD for metallic nanotube enantiomers are rare, the (8,5) enantiomers with CD intensities of +85.9 and −43.4 at M_11_^+^, and the (7,4) enantiomers with +135.2 and −117.9 at M_11_^+^ presented in this contribution (Table S2), appear, to the best of our knowledge, to be the current lead examples for their enrichment.

**Figure 7:**
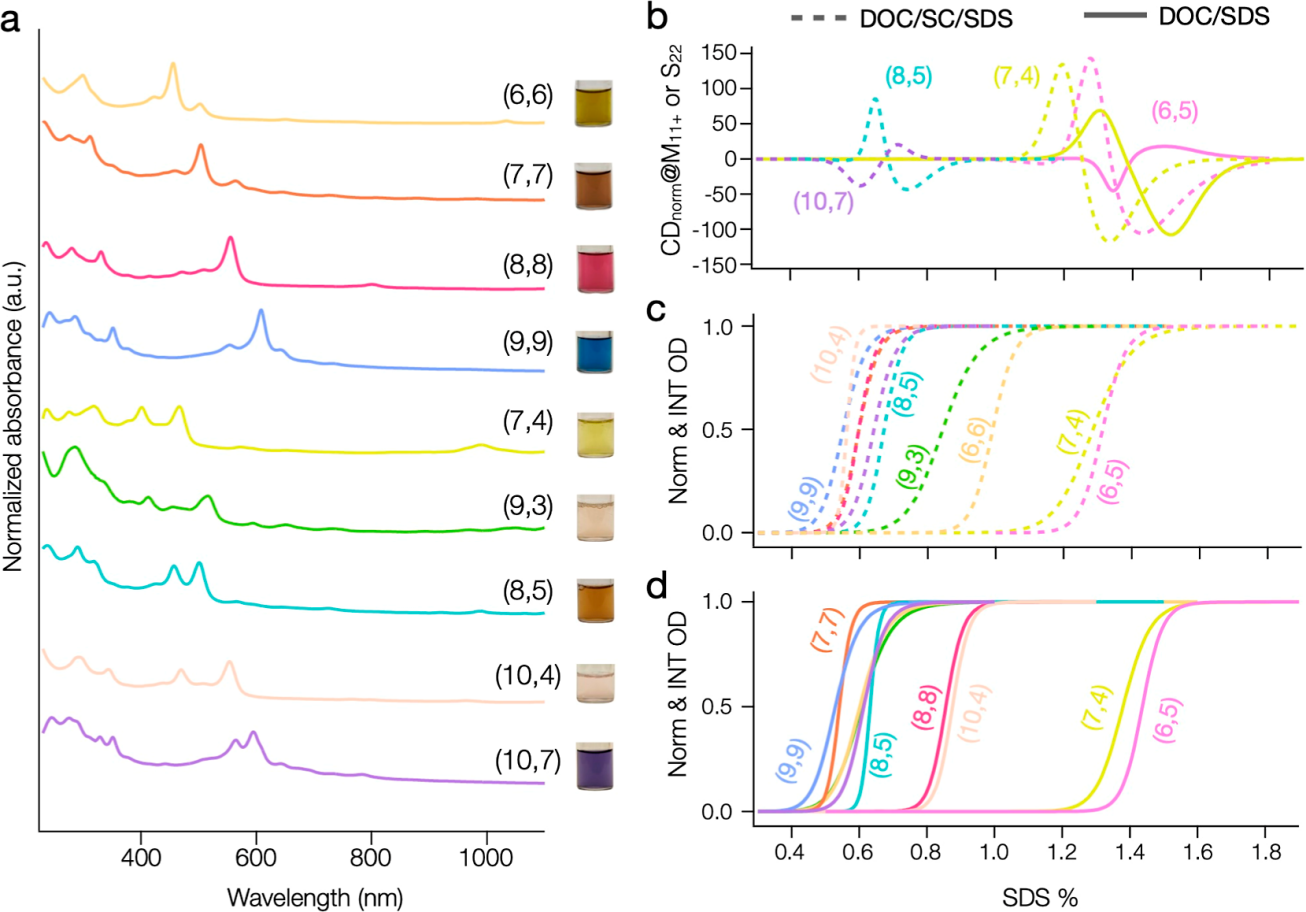
Summary of Sorted Metallic SWCNTs. (a) Absorption spectra and photographs of the obtained metallic (*n*,*m*) species. The spectra are normalized and offset for clear comparison. (b)– (d) Partition curves for the sorted (*n*,*m*) species and their enantiomers. The dashed line indicates separation in the DOC/SC/SDS system, while the solid line represents the DOC/SDS system. The partitioning of (6,5) in the two surfactant systems is also included for comparison.

Figure 7a showcases all the sorted metallic tubes achieved in this study. Peak positions of optical transition for each have been detailed in Table S1. Notably, some nonarmchair species have been separated with high purity. These species, characterized by their relatively low abundance in any raw soot, present significant challenges in their separation. While monochiral sorting of (8,5) and (10,4) has previously been achieved using DNA-assisted methods,^16,44^ (9,3) and (10,7) represent novel species isolated in this study. Remarkably, (10,7) has been separated at the enantiomeric level.

Focusing on Figure 7b–d, the sigmoidal partition curves and the modified Hill equation fitting used for enantiomer separation effectively illustrate the PCCCs for each species under DOC/SDS (7c) and DOC/SC/SDS (7d). On the same SDS x-scale, the distinct positive and negative peaks in Figure 7b show the specific concentrations at which each enantiomer achieves separation in DOC/SDS (solid lines) and DOC/SC/SDS (dashed lines), while the peak heights provide insights into their relative enantiomeric purity (see Table S2 for details). The PCCC values, as graphically reported in Figures 7c,d, are also listed in Table S1. Together, these results clearly demonstrate the advantages of the DOC/SC/SDS ternary system in enhancing the resolution and purity of the sorted species.

## Discussion

In this study, we have demonstrated a systematic approach to precisely determine the PCCCs for various metallic SWCNT species using ATPE with both binary and ternary surfactant systems.

**Figure 8:**
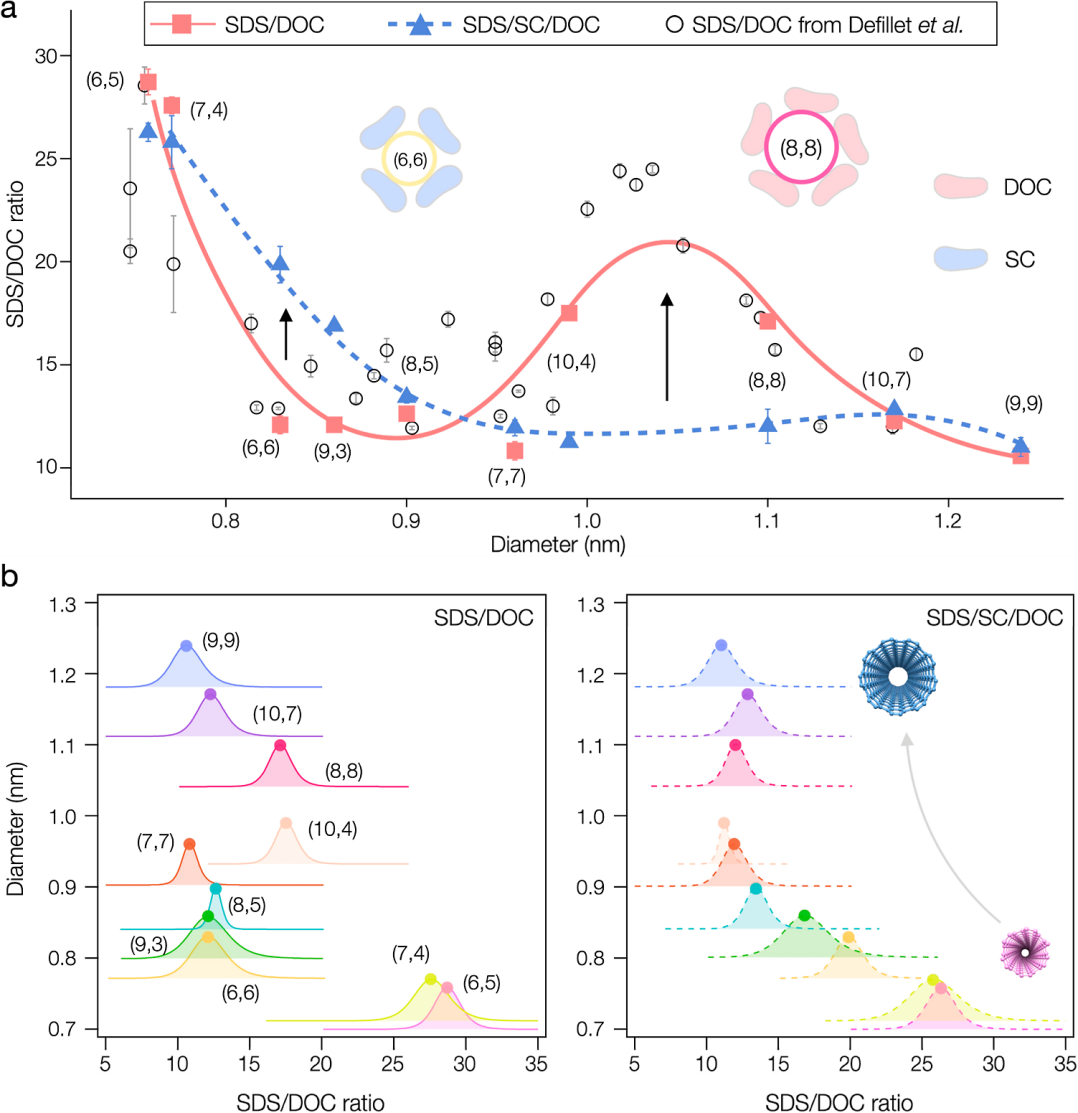
(a) SDS/DOC ratio with 0.05% DOC plotted against SWCNT diameter for ATPE separations in binary (DOC/SDS) systems (red squares) and ternary (DOC/SC/SDS, with 1% SC) systems (blue triangles), compared with data (open circles) from Defillet et al.^28^ Arrows indicate significant deviations in PCCC for tubes like (6,6) and (8,8), highlighting the impact of SWCNT diameter on interactions with different surfactants. (b) Partition curves are represented by Lorentzian-like peaks, showing the PCCC at different SDS/DOC ratios. The peak width, inversely proportional to the Hill coefficient *n*_H_, indicates the range of partitioning for specific SWCNT species. Binary system curves are depicted with solid lines, while the ternary system is shown with dashed lines.

One of the notable observations is the atypical partition behavior of certain diameter SWCNTs, specifically the (6,6) and (8,8) species, which do not strictly follow the typical diameter-dependent sorting order in both surfactant systems. In the binary system (DOC/SDS) the (8,8) tube (carbon centers definition diameter ≈1.1 nm) exhibits a higher PCCC than anticipated based on its diameter. Conversely, in the ternary system (DOC/SC/SDS) the (6,6) tube (diameter ≈0.83 nm) shows a significantly increased PCCC, deviating from the trend observed for other armchair species. Figure 8a illustrates these findings by plotting the SDS/DOC ratio at the PCCC point against the diameter of SWCNTs. Our finding in the binary surfactant system is consistent with the lower precision results from the literature.^29^ Previous research using a series of single-step ATPE^29^ and empirical sequential ATPE results^15^ have shown similar results for the DOC/SDS binary case for larger diameter tubes like (8,8), but without the DOC/SC/SDS ternary case involving smaller diameter tubes like (6,6). We believe that both anomalous behaviors can be attributed to the differential adsorption and packing efficacy of surfactants around specific diameter tubes, which influences their solvation energies and, consequently, their partitioning in the ATPE system.

From previous studies, it is known that the adsorption and wrapping efficacy of DOC and SC on SWCNTs of varying diameters play a crucial role in this context, even though they differ by only one hydroxyl group. While a small difference, this does result in distinct properties for DOC and SC (and other bile salt variant) molecules in solution; one example is that the two molecules have different aggregation numbers when forming micelles in water. DOC has an aggregation number of approximately 3.1 at room temperature, and SC has about 2.7,^45^ with the difference ascribed to hydrogen bonding. While the adsorbed layer of DOC or SC on a SWCNT are expected to be stabilized primarily by interactions with the nanotube surface,^41^ the exact packing confirmation is also strongly affected by the surfactant structure.^23,46^

In this contribution, by focusing on armchair SWCNTs, we eliminate the confounding effects of handedness and enable analysis of the PCCC values through only a diameter-dependence framework. The results of this contribution thus underscore the importance of surfactant–nanotube interactions in determining partitioning outcomes. For example, using SDS/DOC/SC first can help isolate (6,6) from other armchair nanotubes, while the binary SDS/DOC system is better at selectively extracting (8,8). By combining both systems in a multistep extraction, one can tailor the process to address more complex separation requirements. However, to gain a more comprehensive understanding of the wrapping conformations and aggregation numbers of surfactants on different diameter nanotubes, further studies employing techniques like analytical ultracentrifugation (AUC) are desirable. Such investigations could provide quantitative data on the mass of adsorbed surfactants and the structural arrangement of the surfactant–nanotube complexes, offering deeper insights into the mechanisms underlying the observed partitioning behaviors.^46^

The sharp cooperativity observed in the partitioning transitions, as indicated by the high Hill coefficients (*n*_H_) obtained from fitting the partition curves, reflects the highly cooperative nature of surfactant adsorption on the nanotube surfaces, which is consistent with many previous studies.^29,40,41^ This cooperativity is illustrated in Figure 8b, where we present the differential form of the Hill equation applied to our data. In this figure, PCCC values are indicated by the peak positions along the SDS/DOC ratio axis, and the width of each peak is inversely related to *n*_H_. For better separation, these Lorentzian-type peaks need to be well-separated, sharp, and minimally overlapped. Note that the specific widths for each species may be affected by variations in SWCNT length,^47^ defectiveness or filling^21,22^ within a particular population.

This cooperativity is hypothesized to result from the “fish scale” tiling model of bile salt surfactant molecules forming an ordered, overlapping arrangement on the nanotube surface, enhancing the stability of the coating and amplifying changes in solvation energy at specific surfactant concentrations. However, compared to the PCCC values, we observe larger variability in the *n*_H_ values and associated uncertainties, without a clear trend based on diameter or chirality (see Table S1 in the Supporting Information). This suggests that multiple factors—such as enantiomer, length distribution, filling (e.g., with water or other molecules), source variations, and starting suspension conditions—may influence the cooperativity and partitioning behavior.^30,31^

For example, species like (10,7) and (9,9) from FCVD synthesis (TUBALL) as prepared for this contribution are filled with C_24_H_50_. Along with narrower optical transitions attributable to improved homogeneity of their interior environment compared to water-filled SWCNTs, in PCCC determination these SWCNTs exhibit particularly low uncertainties in their *n*_H_ values, which may similarly reflect a reduction in heterogeneity consistent with literature^48,49^ and previously observed for semiconducting species separations.^21^ To delve more deeply into this issue, more accurate sample preparation techniques, such as length sorting and various filling comparisons, will be the focus of future research.

For impacts to separations, we observe that in the SDS/SC/DOC system, most peaks are more dispersed than in the SDS/DOC case (Figure 8b), implying better resolution in most cases. For example, the separation of challenging nonarmchair species like (8,5), (10,4), (9,3), and (10,7) is notably enhanced, with the ternary system facilitating the isolation of high-purity fractions and even enabling enantiomer-level separations.

## Conclusion

In conclusion, this work advances the understanding of surfactant-controlled partitioning mechanisms in ATPE for metallic SWCNTs and their enantiomers. By precisely determining the PCCCs in both binary and ternary surfactant systems, we highlight the critical role of surfactant–nanotube interactions and cooperativity in achieving high-resolution separations. The findings underscore the importance of selecting appropriate surfactants to modulate solvation energies effectively and suggest that further studies, including detailed characterization of surfactant coatings and exploration of other surfactant combinations, could lead to even more efficient and precise separation methodologies. Future work involving techniques like AUC and molecular simulations could provide deeper insights into the surfactant assembly on nanotube surfaces, paving the way for the rational design of surfactant systems tailored for specific separation challenges in nanomaterials science.

## Experimental Methods

Certain equipment, instruments, software, or materials, commercial or noncommercial, are identified in this paper in order to specify the experimental procedure adequately. Such identification is not intended to imply recommendation or endorsement of any product or service by NIST, nor is it intended to imply that the materials or equipment identified are necessarily the best available for the purpose.

### Presorted SWCNT Sample Preparation

Three types of SWCNT raw soots were utilized to prepare various (*n*,*m*) species: CoMoCat SG65i (Chasm Nanotechnologies or via Sigma-Aldrich), HiPco (NoPo Nanotechnologies, India), and TUBALL (OCSiAl). Initially, each type of raw material, containing 30 mg of SWCNT soot in 30 mL of 10 g/L (1%, as stated in the main text, all % values listed are mass/volume) sodium deoxycholate (DOC, BioChemica), was subjected to 45 min of tip sonication (Ultrasonic Homogenizer, FS-750T) in an ice bath, followed by 1 h of centrifugation at 16,639*g* (Eppendorf 5810). Based on earlier protocols,^23^ a rough SDS cut was applied for diameter sorting and semiconducting-metallic separation using an ATPE system composed of Poly(ethylene glycol) (PEG, MW 6 kDa, Sigma-Aldrich) and Dextran (MW 70 kDa, TCI). Specifically, for the CoMoCat material, a diameter sorting procedure was implemented with a constant 0.05% DOC, alongside two SDS concentrations of 1% and 1.5%. Two top phases, T1 and T2, were collected at these respective SDS concentrations for further semiconducting-metallic separation. In next steps, the overall surfactant concentrations were adjusted to 0.9% sodium cholate (SC, Sigma-Aldrich), 1% SDS, and less than 0.02% DOC. To aid in the semiconducting-metallic separation, sodium hypochlorite (NaClO, (10–15) % available chlorine, Honeywell) was added at 5 μL/mL, prediluted to a 1/100th concentration in water. The metallic-enriched fractions from T1 were further processed for (6,6) and (9,3) separation, while the metallic fraction from T2 was used for (7,4) and its enantiomer sorting. The semiconducting fraction of T2 was used for the sorting of (6,5). For HiPco material, similar procedures were followed but with different SDS concentrations: 0.7% SDS for (7,7) and (8,5), and 1% SDS for (8,8) and (10,4). Metallic TUBALL starting material was prepared as previously reported.^50^ TUBALL soot was incubated with C_24_H_50_ at 60 °C for 24 h, above the alkane’s melting point, to fill the nanotubes.^49^ Following the filling process, the material was filtered and rinsed with heptane to remove excess alkane, resulting in C_24_H_50_@TUBALL. The filled SWCNT powder was then dispersed in a 2% DOC aqueous solution and ultrasonicated, followed by centrifugation to remove impurities and collect the supernatant. Rate-zonal ultracentrifugation was performed using a 10% iodixanol density gradient to isolate structurally pristine, individualized nanotubes as in previous reports.^51^ The purified nanotubes underwent ATPE to enrich metallic species,^52^ ensuring high purity and uniformity in the final sample. All presorted samples were reconcentrated and adjusted to 1% DOC (m/v) in a pressurized ultrafiltration stirred cell (Millipore) equipped with a 300 kDa cutoff membrane for further PCCC measurements.

### Further ATPE Separation for PCCC Identification

The presorted samples, already enriched with specific species, were used to precisely identify the PCCCs for all armchair and nonarmchair SWCNTs. Following our previously reported methodology,^23^ we typically used 5 mL each of the bottom and top phases from the ATPE system to conduct the separation. In the binary cosurfactant system, DOC concentration was maintained at 0.05%, and SDS concentration was gradually increased to facilitate the migration of SWCNTs to the top phase. In the ternary system, concentrations of DOC and SC were fixed at 0.05% and 1%, respectively. A 5 mL mimic top phase was added iteratively for sequential extractions. This process involved adding a new mimic phase, obtaining a fraction, extracting this fraction, and then introducing a new mimic phase at a higher SDS concentration to achieve the next fraction. To ensure a consistent and good yield of carbon nanotubes in the top phase and to standardize the procedure for easier comparison, each increment in SDS concentration was set at either 0.05% or 0.1%. In the end, all sorted fractions from the extracted top phase were reconcentrated and adjusted to 1% DOC (mass/volume) in a pressurized ultrafiltration stirred cell (Millipore) with a 300 kDa cutoff membrane for subsequent spectroscopic characterizations. All ATPE separations were performed at room temperature (21–22 °C).

### Absorption and CD Measurement

UV–Vis–NIR absorbance spectra were collected on a SPECORD 250 PLUS spectrometer from (200 to 1100) nm for all samples in cuvettes with 10 mm path length. CD measurements were performed on a CD spectrometer (Chirascan-plus, Applied Photophysics) from (800 to 200) nm through a 10 mm path length cuvette (step size 1 nm; bandwidth 2 nm).

### Modified Hill Equation for PCCC of Enantiomer Fitting

To analyze the CD data for enantiomer sorting, we employed a modified Hill equation, tailored to accommodate the separation of two distinct types of enantiomers. The conventional Hill equation used for fitting normalized OD data is represented by eq 1. To adapt this model for CD analysis, where two enantiomers exhibit distinct separation behaviors, the equation was differentiated and divided into two components. Each component represents one enantiomer
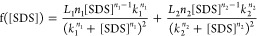
2Here, [SDS] denotes the SDS concentration, while *L*_1_ and *L*_2_ are coefficients corresponding to the CD signal peak strength of each enantiomer. *k*_1_ and *k*_2_, also defined as PCCC from eq 1, describe the SDS concentration at which the enantiomer transition is maximized. The exponents *n*_1_ and *n*_2_ represent the Hill coefficients, which indicate the cooperativity of SDS binding to each enantiomer.

## Supporting Information Available

The following files are available free of charge. Detailed absorption spectra data throughout the sorting process for single-chirality SWCNTs; CD spectra for the enantiomer sorting and a summary of peak intensities; summary of PCCC values (PDF)
