# Supplementary material for: Precise Partitioning of Metallic Single-Wall Carbon Nanotubes and Enantiomers through Aqueous Two-Phase Extraction
Source: ACS Nano. 2025 Apr 3;19(14):14137–49. doi: 10.1021/acsnano.5c00025 (PMC12005050; doi:10.1021/acsnano.5c00025)
Supplement: Supplementary file 1 — nn5c00025_si_001.pdf [file nn5c00025_si_001.pdf]

SUPPORTING INFORMATION

Precise Partitioning of Metallic Single-Wall Carbon Nanotubes and Enantiomers Through Aqueous Two-Phase Extraction

Han Li[1, 2 *], *Ming Zheng*[3], *Jeffrey A. Fagan*[3 *]

1. Department of Mechanical and Materials Engineering, University of Turku, FI-20014 Turku, Finland

2. Turku Collegium for Science, Medicine and Technology, University of Turku, FI-20520 Turku, Finland

3. Materials Science and Engineering Division, National Institute of Standards and Technology, Gaithersburg, MD, 20899, USA

*corresponding authors: han.li@utu.fi, jeffrey.fagan@nist.gov

Certain equipment, instruments, software, or materials, commercial or non-commercial, are identified in this paper in order to specify the experimental procedure adequately. Such identification is not intended to imply recommendation or endorsement of any product or service by NIST, nor is it intended to imply that the materials or equipment identified are necessarily the best available for the purpose.

Uncertainties are reported as one standard deviation (σ) unless otherwise noted.

Acronyms

Single-Wall Carbon Nanotubes, SWCNTs
Aqueous Two-Phase Extraction, ATPE
Sodium deoxycholate, DOC
Sodium cholate, SC
Sodium dodecyl sulfate, SDS
Ultraviolet-visible-near infrared, UV-vis-NIR
Circular dichroism, CD
Partition coefficient change condition, PCCC
Cobalt-molybdenum catalyst, CoMoCat
High pressure CO disproportionation, HiPCo
Floating catalytic vapor deposition, FCVD

NOTE:

Surfactant concentrations are reported in mass/volume percentages in accordance with typical literature practice.

Figures and Tables:

Figure S1 | Absorbance spectra of the presorted samples 2

Figure S2 | Absorption spectra of (6,6), (7,7), (8,8) and (9,9) sorting in DOC/SDS 4

Figure S3 | Absorption spectra of (6,6), (7,7), (8,8) and (9,9) sorting in DOC/SC/SDS 5

Figure S4 | (8,5) sorting in binary and ternary surfactant systems 6

Figure S5 | (10,7) enantiomer sorting in DOC/SC/SDS 7

Figure S6 | (10,7) sorting in binary and ternary surfactant systems 8

Figure S7 | CD spectra of (7,4) enantiomer sorting in binary and ternary surfactant systems ..9

Figure S8 | Absorption spectra of (7,4) sorting in binary and ternary surfactant systems 10

Table S1 | Summary of PCCCs and Hill coefficient (nH) for the sorted single-chirality SWCNTs. 11

Table S2 | Summary of enantiomer PCCCs for the sorted non-armchair metallic SWCNTs..12

Figure S1 | Absorbance spectra of the metallic species-enriched presorted samples from different SWCNT soot sources.

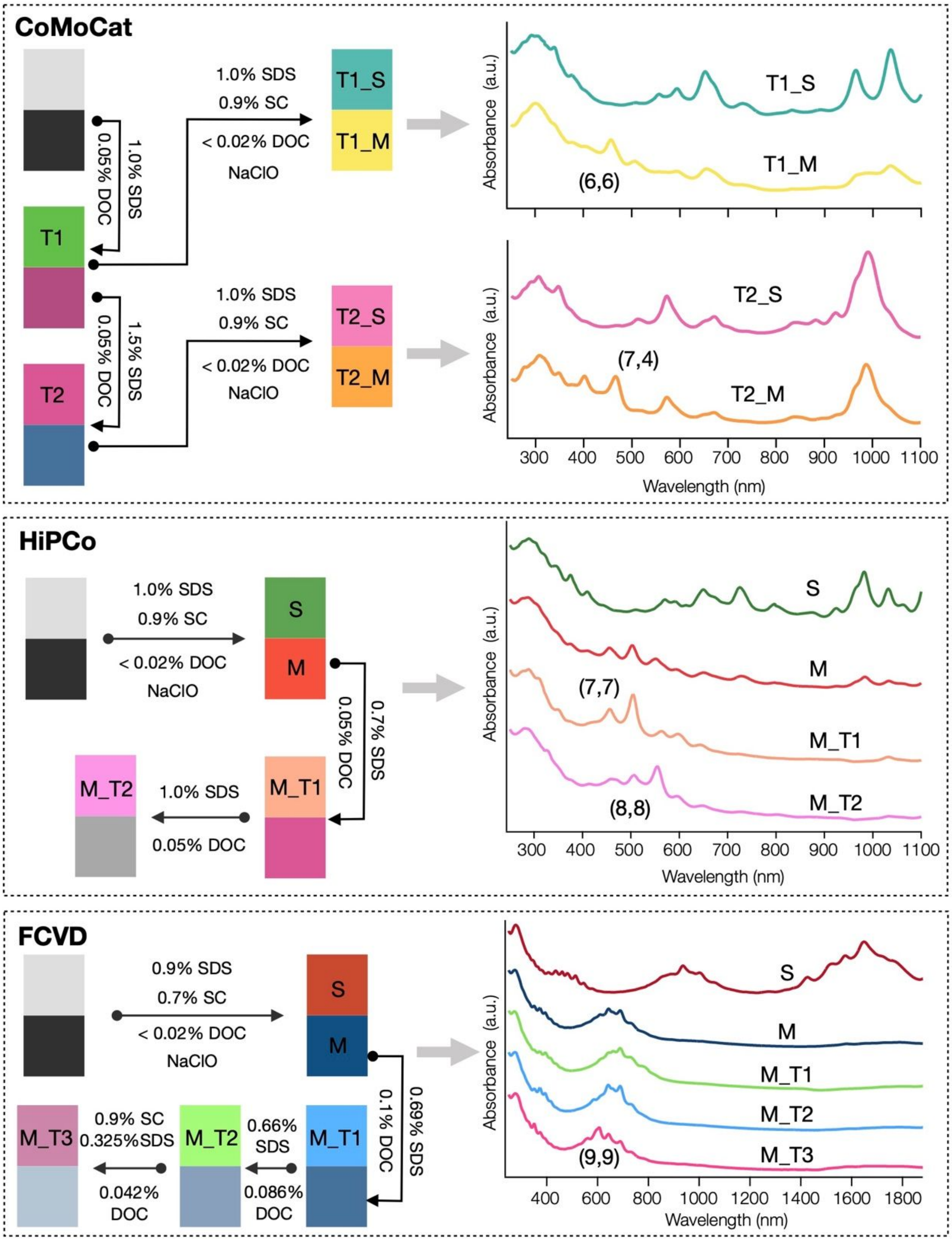

Figure S1. Detailed procedures and absorbance spectra of the presorted samples used as starting materials for further separation. Enriched (6,6) and (7,4) are from CoMoCat SG65i-type raw material; (7,7) and (8,8) originate from NoPo (HiPCo synthesis method) raw soot; (9,9) is from TUBALL (FCVD method) with $C_{24}H_{50}$ filling. Detailed experimental procedures related to the sample preparation and sorting are also described in the Methods section.

Figure S2 | Absorbance spectra of (6,6), (7,7), (8,8) and (9,9) sorting in DOC/SDS

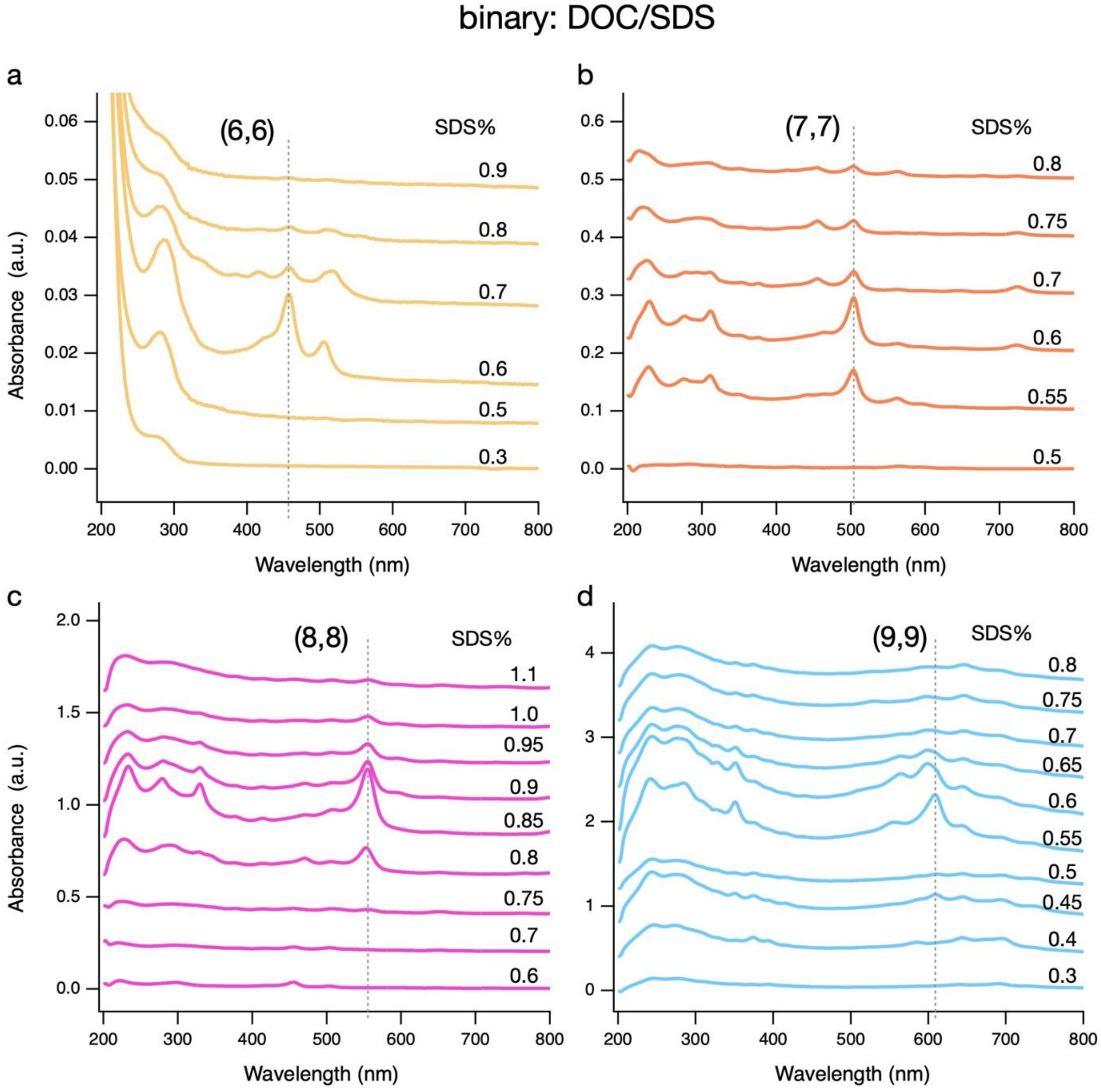

Figure S2. Absorbance spectra of each step through the sorting process of (6,6), (7,7), (8,8) and (9,9) in the binary cosurfactant system DOC/SDS. In all experiments the DOC concentration was maintained at 0.05 %. SDS concentrations for each fraction are marked on the graph for each spectrum. The vertical dashed line reports the peak position of the major species being tracked. All measures are conducted using a cuvette of 10 mm path length, with a mimic top phase used as the background. The spectra are offset for clearer comparison.

Figure S3 | Absorbance spectra of (6,6), (7,7), (8,8) and (9,9) sorting in DOC/SC/SDS

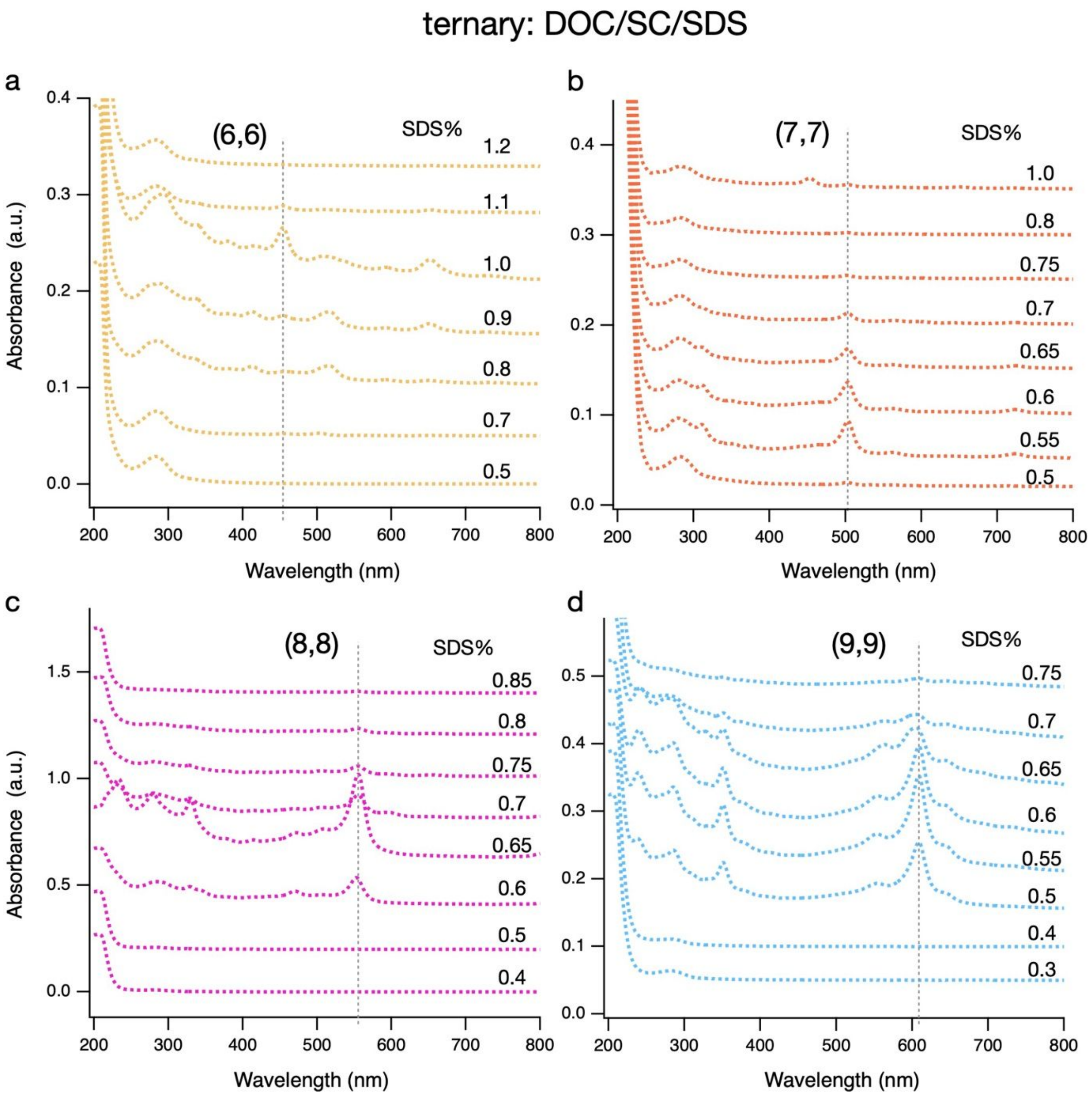

Figure S3. Absorbance spectra of each step through the sorting process of (6,6), (7,7), (8,8) and (9,9) in the ternary cosurfactant system DOC/SC/SDS. DOC concentration is maintained at 0.05 %, and SC at 1 % for all experiments. SDS concentrations for each fraction are marked on the graph for each spectrum. The vertical dashed line reports the peak position of the major species being tracked. All measures are conducted using a cuvette of 10 mm path length, with a mimic top phase used as the background. The spectra are offset for clearer comparison.

Figure S4 | (8,5) sorting in binary and ternary surfactant systems

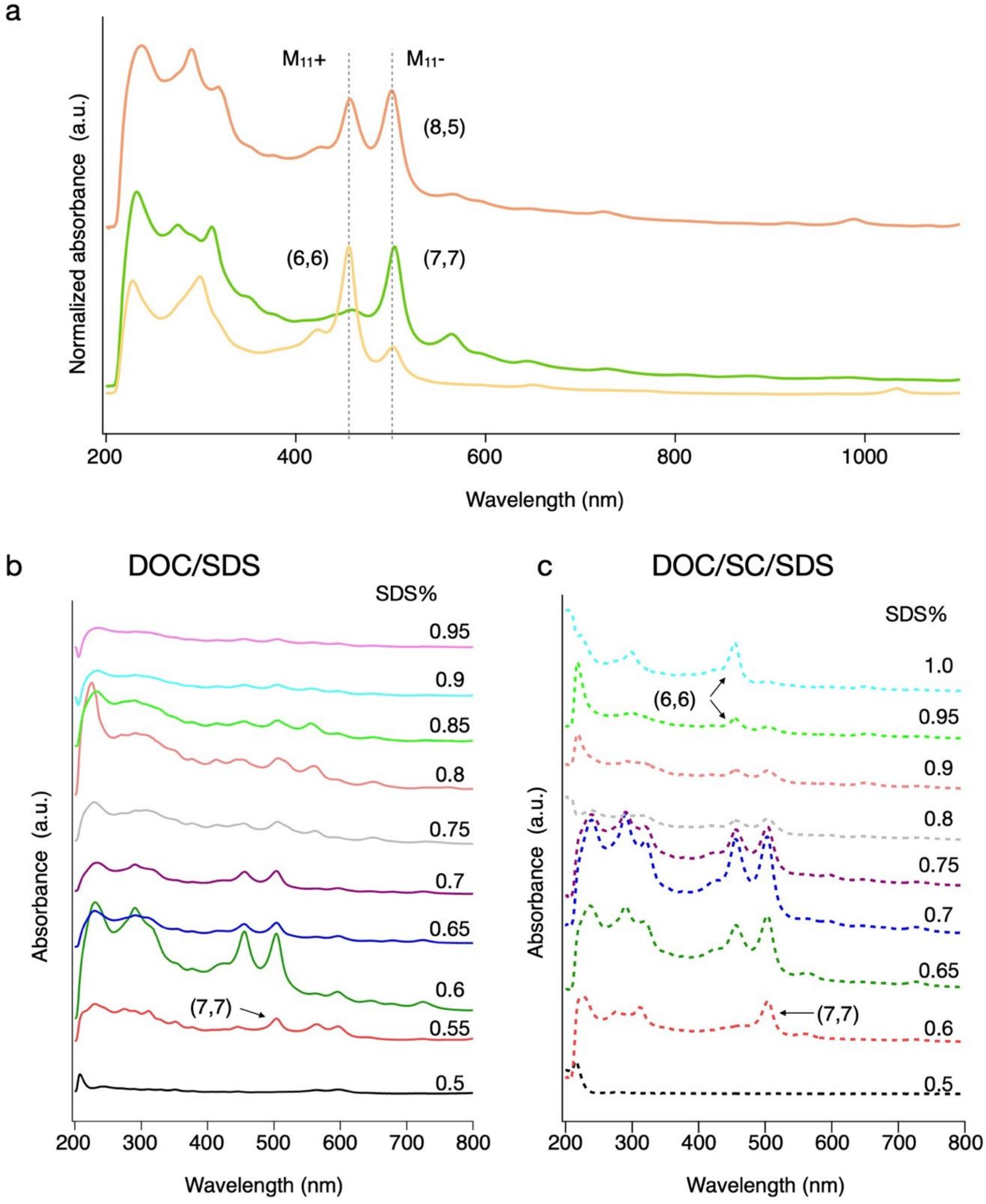

Figure S4. (a) The overlapping M_{11} peaks of (6,6) and (7,7) with M_{11}+ and M_{11}- of (8,5) respectively. (b) Absorbance spectra at each step in the sorting process of (8,5) using DOC/SDS, with a constant DOC concentration 0.05 %. (c) Absorption spectra through the sorting process of (8,5) in DOC/SC/SDS cosurfactant systems, with a constant DOC at 0.05 % and SC at 1 %. SDS concentrations for each fraction are marked on the graph. The ternary system shows improved resolution, allowing (7,7) and particularly (6,6) at around 1 % SDS to be separated more effectively (indicated by the arrows).

Figure S5 | (10,7) enantiomer sorting in DOC/SC/SDS

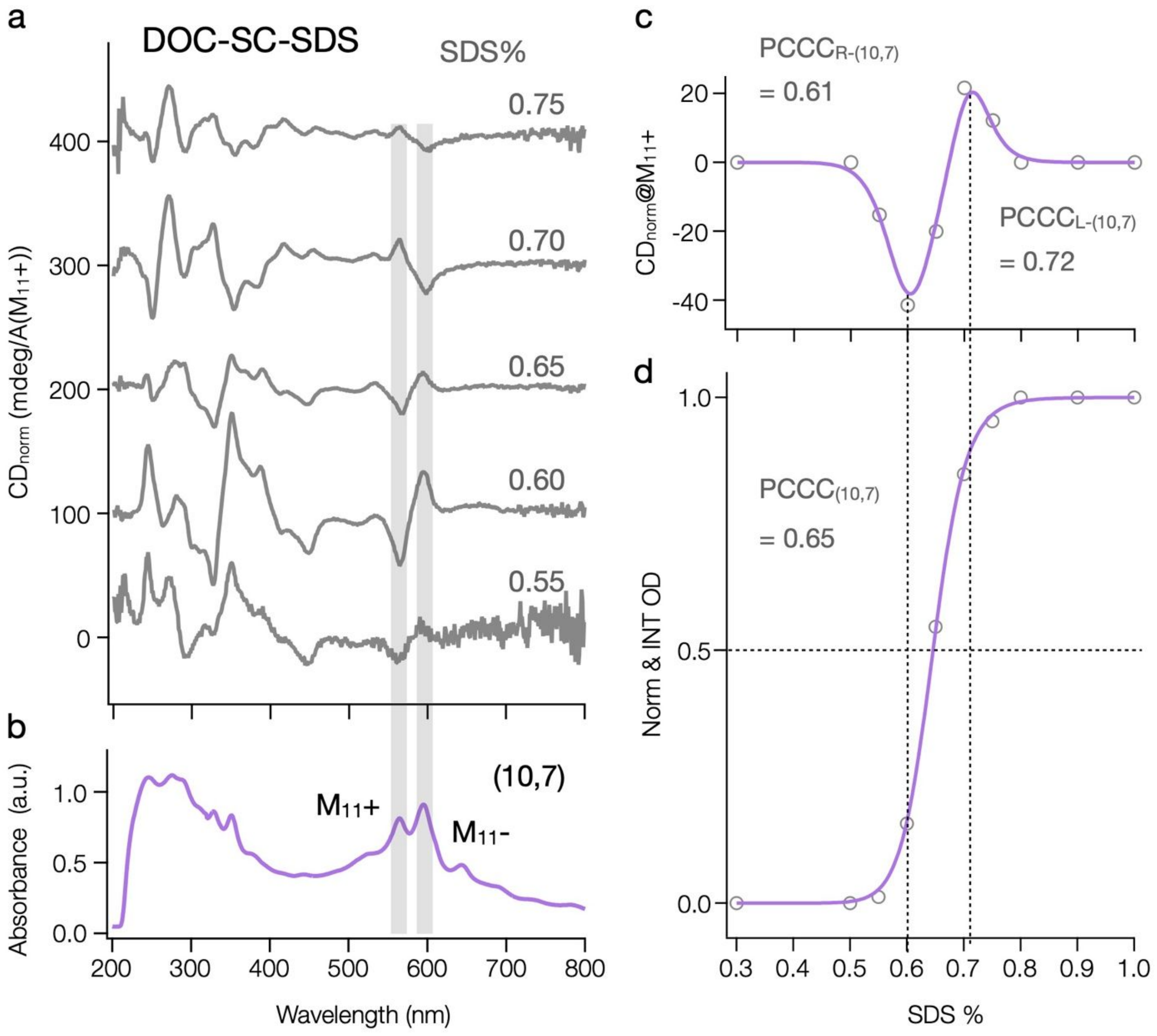

Figure S5. Enantiomer separation of (10,7) in the DOC/SC/SDS system. (a) CD spectra for (10,7) enantiomers at increasing SDS concentrations from 0.55 % to 0.75 %, normalized by the M_{11}+ peak OD values from the absorption spectra. (b) A typical absorbance spectrum of (10,7) showcasing both the M_{11}+ and M_{11}- peak positions, corresponding to the plus and minus CD peaks in (a). (c) Normalized CD values at the M_{11}+ are plotted against SDS concentration and fitted using a modified Hill equation in differentiated form, indicating the enantiomer PCCCs. (d) The corresponding partition curve for (10,7) demonstrates the single-chirality PCCC. All absorbance and CD measurements were conducted in 1 % DOC in H_2O. Spectra are offset for clearer comparison.

Figure S6 | (10,7) sorting in binary and ternary surfactant systems

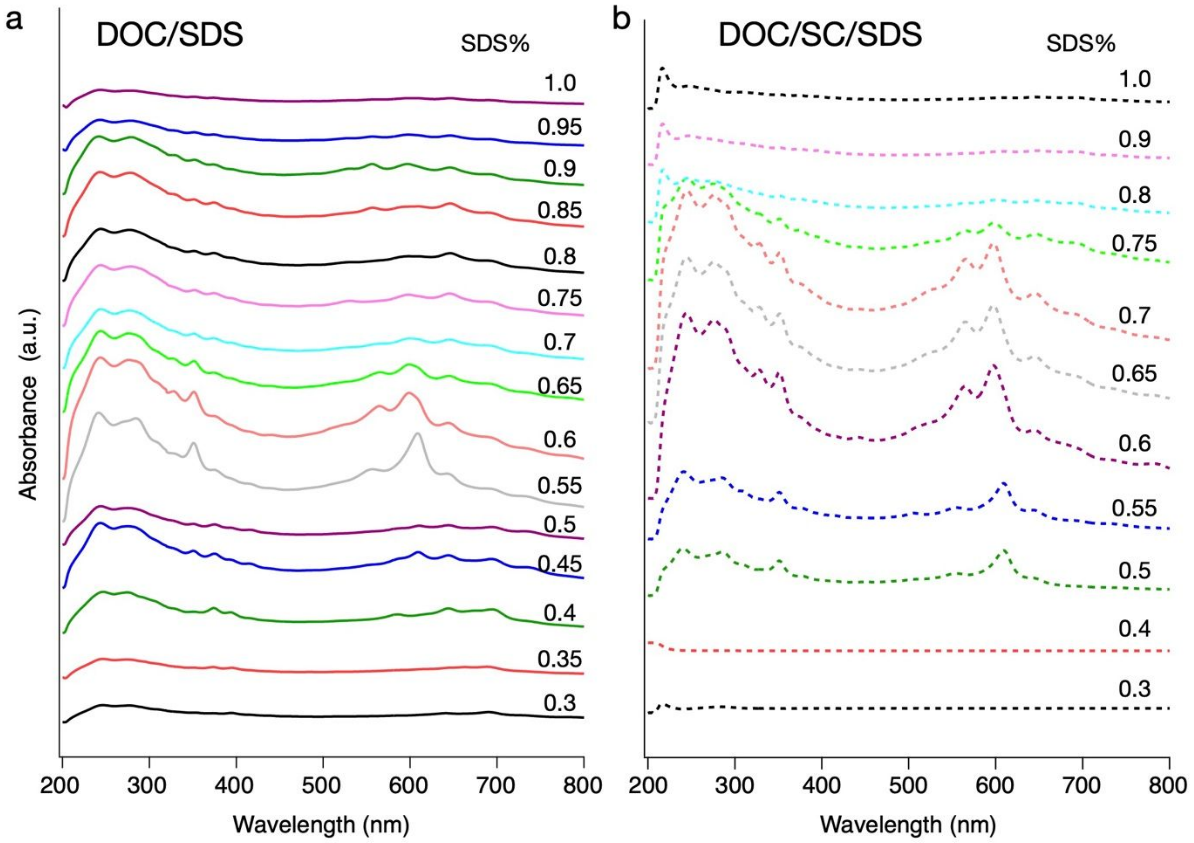

Figure S6. (a) Absorbance spectra at each step in the sorting process of (10,7) using DOC/SDS, with a constant DOC concentration 0.05 %. (c) Absorption spectra through the sorting process of (10,7) in DOC/SC/SDS cosurfactant systems, with a constant DOC at 0.05 % and SC at 1 %. SDS concentrations for each fraction are marked on the graph. The ternary system shows improved resolution, showing better (10,7) and (9,9) separation.

Figure S7 | CD spectra of (7,4) enantiomer sorting in binary and ternary surfactant systems

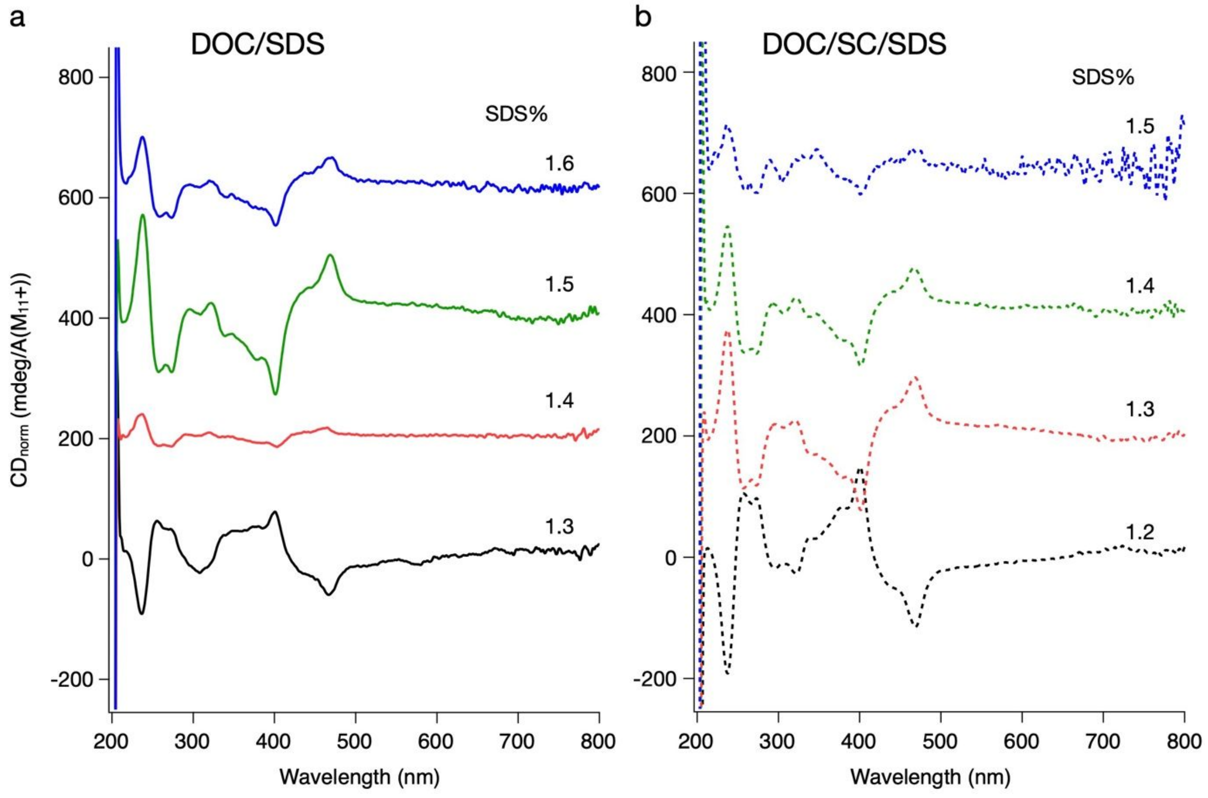

Figure S7. Normalized CD spectra of (7,4) enantiomers separated in (a) the DOC/SDS cosurfactant system and (b) DOC/SC/SDS system. SDS concentrations for each fraction are marked on the graph. The spectra are offset by 200 mdeg/A increments for clearer comparison.

Figure S8 | Absorbance spectra of (7,4) sorting in binary and ternary surfactant systems

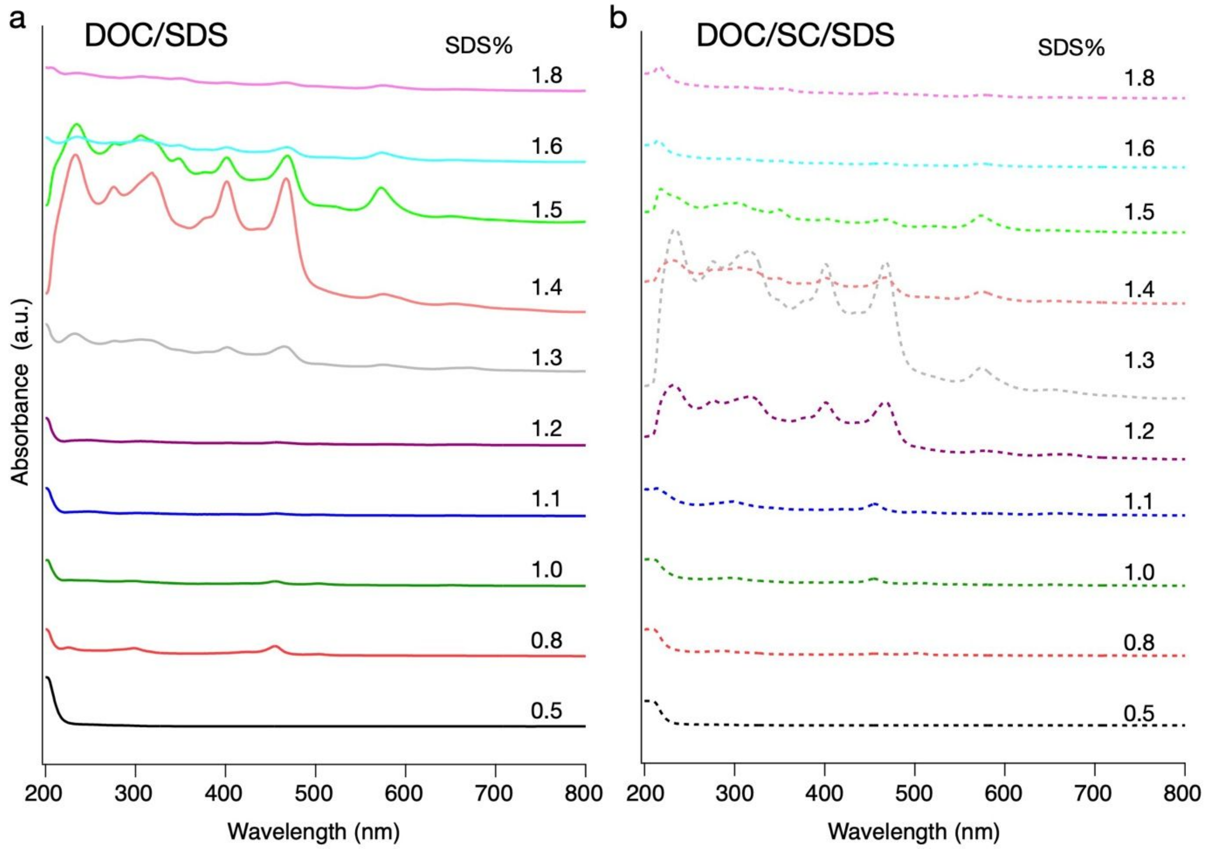

Figure S8. Absorbance spectra at each step in the sorting process of (7,4) using (a) DOC/SDS cosurfactant, with a constant DOC concentration 0.05 %, and (b) DOC/SC/SDS cosurfactant systems, with a constant DOC at 0.05 % and SC at 1 %. SDS concentrations for each fraction are marked on the graph.

Table S1 | Summary of PCCCs and Hill coefficient (n_H) for the sorted single-chirality SWCNTs.

**(n,m)**	**Diameter (nm)**	**Optical Transition Peak (in 1% DOC / H_2O)**		**DOC/SDS**				**DOC/SC/SDS**			
		Type	Wavelength (nm)	PCCC (%)	σ_{PCCC} (%)	n_H	σ_{n_H}	PCCC (%)	σ_{PCCC} (%)	n_H	σ_{n_H}
**(6,5)**	0.76	S_{11}	991	1.43	0.03	48.1	4.5	1.31	0.02	38.9	5.1
**(6,6)**	0.83	M_{11}	456	0.60	0.02	16.9	6.6	0.99	0.04	42.9	11.3
**(7,4)**	0.77	$M_{11}+$	401	1.38	0.02	34.2	5.3	1.29	0.06	27.3	6.3
		$M_{11}-$	466								
**(7,7)**	0.96	M_{11}	504	0.54	0.02	37.2	2.7	0.60	0.02	22.6	6.5
**(8,5)**	0.90	$M_{11}+$	457	0.63	-	48.4	-	0.67	0.01	27.6	6.3
		$M_{11}-$	501								
**(8,8)**	1.10	M_{11}	555	0.86	0.02	36.9	9.6	0.60	0.04	43.6	11.0
**(9,3)**	0.86	$M_{11}+$	413	0.60	-	13.6	-	0.84	-	16.1	-
		$M_{11}-$	516								
**(9,9)**	1.24	M_{11}	608	0.53	0.01	15.2	1.5	0.55	0.02	18.2	3.2
**(10,4)**	0.99	$M_{11}+$	470	0.87	-	35.4	-	0.56	0	46.6	-
		$M_{11}-$	554								
**(10,7)**	1.17	$M_{11}+$	565	0.61	-	19.4	-	0.64	0.01	20.3	2.8
		$M_{11}-$	595								

The uncertainties associated with the PCCC (in % SDS) and n_H values (unitless) were assessed to understand the variability and reliability of our experimental data across different surfactant conditions and SWCNT species. PCCC values demonstrated notable stability across repeated experiments, which shows the robustness of the ATPE and the consistent behavior of surfactant interactions under controlled experimental conditions. An estimated error for PCCC in single-experiment samples can be conservatively set at ± 0.05 %.

In contrast, the n_H exhibited more significant variability, influenced by multiple factors such as SWCNT enantiomer type, length distribution, filling (with substances like water or other molecules), source material variations, and SWCNT composition in initial suspension. These factors can affect the cooperativity of surfactant binding and the subsequent partitioning behavior, leading to differences in the steepness of the partition curves represented by n_H.

Table S2 | Summary of enantiomer PCCCs for the sorted non-armchair metallic SWCNTs.

(n,m)	DOC/SDS				DOC/SC/SDS			
	R		L		R		L	
	PCCC (%)	CD_{norm} mdeg/A(x)	PCCC (%)	CD_{norm} mdeg/A(x)	PCCC (%)	CD_{norm} mdeg/A(x)	PCCC (%)	CD_{norm} mdeg/A(x)
**(6,5)**	1.34	-45.5 (S_{22})	1.49	17.9 (S_{22})	1.43	-88.1 (S_{22})	1.28	106.8 (S_{22})
**(7,4)**	1.51	-107.9 (M_{11}^{+})	1.31	69.2 (M_{11}^{+})	1.34	-117.9 (M_{11}^{+})	1.20	135.2 (M_{11}^{+})
**(8,5)**	-	-	-	-	0.74	-43.4 (M_{11}^{+})	0.65	85.9 (M_{11}^{+})
**(10,7)**	-	-	-	-	0.61	-38.1 (M_{11}^{+})	0.72	20.3 (M_{11}^{+})
